# Conformational Analysis of Thioether Musks Using Density Functional Theory

**DOI:** 10.3390/ijms10083488

**Published:** 2009-08-04

**Authors:** William N. Setzer

**Affiliations:** Department of Chemistry, University of Alabama in Huntsville, Huntsville, AL 35899, USA; E-Mail: wsetzer@chemistry.uah.edu; Tel. +1-256-824-6519; Fax: +1-256-824-6349

**Keywords:** conformational analysis, thioether, musk, macrocycle, density functional theory, *ab initio* molecular orbital theory

## Abstract

A conformational analysis of nine macrocyclic thioether musks has been carried out using molecular mechanics (MMFF), density functional theory (DFT) using both B3LYP and M06 functionals, as well as Hartree-Fock and post-Hartree-Fock (MP2) *ab initio* methods. 6-Thia-, 10-thia- and 4-methyl-5-thia-14-tetradecananolide, 4-thia-, 7-thia-, 11-thia- and 12-thia-15-pentadecanolide and 6-thia- and 12-thia-16-hexadecanolide were modeled. Unfortunately, there was little agreement between the computational methods at the levels of theory used in this study.

## 1. Introduction

Discrepancies in energy differences between density functional (B3LYP) and post Hartree-Fock (MP2) *ab initio* methods have been noted in large, conformationally mobile ring systems, including mesocyclic hydrocarbons [1,2] and macrocyclic sesquiterpenes [3]. In addition, the B3LYP functional has been found to give increased errors with increasing molecular size [4,5], and Schreiner and co-workers [4] have recommended using higher level (*e.g.*, MP2 with a 6–31G** basis set) single-point energy calculations on DFT structures as a confirmation. In order to compare DFT methods with post-HF methods on conformationally mobile macrocycles, a conformational analysis of thioether musks has been carried out.

Naturally occurring musks and their analogs are macrocyclic ketones (*e.g.*, muscone, exaltone, and civetone) or lactones (*e.g.*, muscolide and ambrettolide) [6,7]. These macrocyclic compounds may also possess other functional groups such as ether (–O–), thioether (–S–) or alkene (–C=C–) functionalities [8,9]. In this report, conformational analyses of macrocyclic lactones containing a thioether moiety: 6-thia-14-tetradecanolide (**1**), 10-thia-14-tetradecanolide (**2**), 4-methyl-5-thia-14-tetradecananolide (**3**), 4-thia-15-pentadecanolide (**4**), 7-thia-15-pentadecanolide (**5**), 11-thia-15-pentadecanolide (**6**), 12-thia-15-pentadecanolide (**7**), 6-thia-16-hexadecanolide (**8**), and 12-thia-16-hexadecanolide (**9**), have been carried out using molecular mechanics (MMFF), and *ab initio* (DFT and MP2) methods. The presence of a suitably placed thioether has been found to intensify the odor of macrocyclic lactone musks [6,9].

## 2. Results and Discussion

A Monte-Carlo molecular mechanics conformational search was carried out on each thioether macrocyclic lactone using the MMFF force field [10]. From the molecular mechanics conformational search, those conformations with relative energies (*E*_rel_, calculated energies relative to the lowest energy conformation) ≤ 3 kcal/mol were investigated using density functional theory (DFT) employing the popular hybrid B3LYP functional [11,12] and the 6–31G* basis set, as well as the recently developed M06 combination functional [13] and the 6–31G* basis set. The B3LYP functional was chosen because it is the popular choice for modeling organic compounds, the M06 functional was chosen because it was developed to predict accurate structures and energies of main-group-containing compounds and includes concovalent interactions. The 6–31G* basis set was chosen for its relatively rapid calculations. In order to confirm the energies from the B3LYP and M06 analyses, single-point Hartree-Fock (HF), followed by second-order Møller-Plesset electron correlation (MP2) calculations at the 6–31G** level were carried out using the B3LYP geometries [4].

### 2.1. 6-Thia-14-tetradecanolide (**1**)

**Figure 1.**
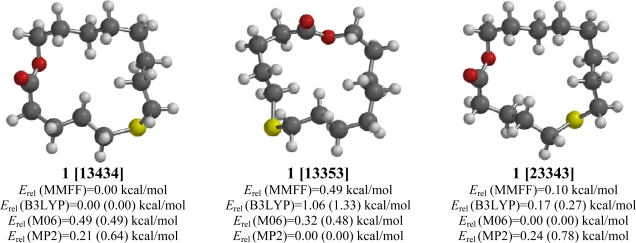
Low-energy conformations of 6-thia-14-tetradecanolide (**1**). Values in parentheses are with diffuse basis sets (6–31+G* for B3LYP and M06, 6–311+G** for MP2).

6-Thia-14-tetradecanolide (**1**) had 30 conformations that had *E*_rel_ (MMFF) ≤ 3.0 kcal/mol. The lowest-energy conformation, [13434], was also the lowest-energy conformation from the B3LYP analysis (see Figure 1). *Note*: macrocyclic conformations are designated according to the system of Dale [14]. In square brackets are indicated the number of bonds in the *trans-*configured edges of the macrocycle, starting with the shortest *trans-*chain, and progressing in the direction of the next shortest; the sum of the numbers in the square bracket is equal to the ring size. An alternative conformation, [13353], however, was the lowest-energy structure according to the MP2 calculations. The M06 calculations indicated a [23343] conformation to be lowest in energy, but this conformation was also very low in energy in the other three computational methods. The lowest-energy conformation for cyclopentadecane using molecular mechanics has been found to be the quinquangular [33333] conformation [14], but an X-ray crystal structure of cyclopentadecanone (exaltone) revealed a [13353] conformation [15]. The lowest-energy [33333] conformation was 1.15, 1.98, and 1.03 kcal/mol higher in energy than the respective lowest-energy conformations for MMFF, M06, and MP2, but only 0.37 kcal/mol higher than the [13434] for B3LYP. In each of the low-energy conformations ([13434], [13353], and [23343]) the thioether moiety can adopt a preferred gauche C-S-C-C torsion angle [16,17]. Additionally, the ester group adopts a preferred *s-trans* orientation [18–20] in each of these conformations.

### 2.2. 10-Thia-14-tetradecanolide (**2**)

**Figure 2.**
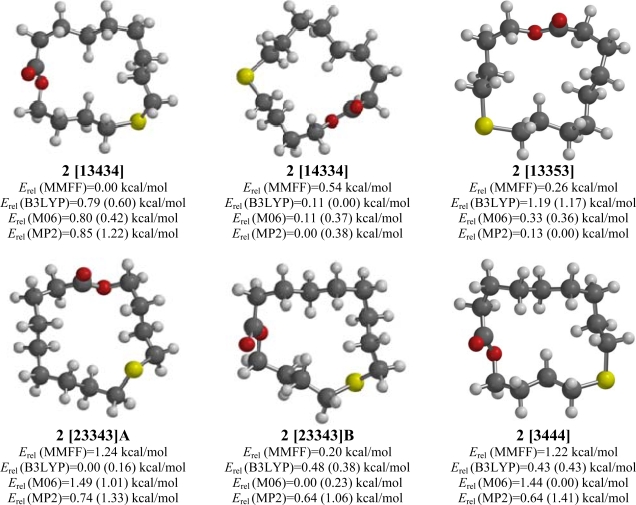
Low-energy conformations of 10-thia-14-tetradecanolide (**2**). Values in parentheses are with diffuse basis sets (6–31+G* for B3LYP and M06, 6–311+G** for MP2).

10-Thia-14-tetradecanolide (**2**) also showed disagreement between the computational methods. There were 32 low-energy conformations from the MMFF calculations, of which a [13434] was the lowest-energy from the MMFF analysis, but a [23343] conformation was shown to be the B3LYP lowest-energy conformation while an alternative [23343] conformation was favored by M06 (Figure 2). The MP2 calculations indicated a fourth conformation, a [14334] conformation, to be the lowest energy form. A [13353] conformation was also low in energy. Although the C-S-C-C groups are gauche in each of these conformations, only in the [14334] and [13353] conformations do the sulfur atom adopt an exodentate “corner” position [21,22]. Similar to what was found for **1**, the lowest-energy [33333] conformation is 1.44, 1.15, and 1.47 kcal/mol higher in energy than the respective lowest-energy conformations for MMFF, M06, and MP2, but only 0.65 kcal/mol higher than the [23342] for B3LYP.

### 2.3. 4-Methyl-5-thia-14-tetradecananolide (**3**)

**Figure 3.**
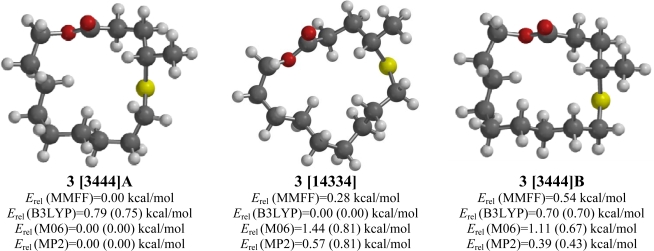
Low-energy conformations of 4-methyl-5-thia-14-tetradecananolide (**3**). Values in parentheses are with diffuse basis sets (6–31+G* for B3LYP and M06, 6–311+G** for MP2).

The steric demands of the methyl group on 4-methyl-5-thia-14-tetradecananolide (**3**) seem to have reduced the number of low-energy conformations (MMFF) to 19. A [3444] (**A**) conformation was the lowest-energy conformation for MMFF, M06, and MP2 (Figure 3). In this conformation, the C-S-C-C unit is *anti* (and endodentate). The B3LYP method, on the other hand, predicted a [14334] conformation. Notably, an alternative [3444] (**B**) conformation was also low in energy, but not the lowest in any of the computational methods. The lowest-energy [33333] conformation was, by MMFF, 3.29 kcal/mol higher in energy than [3444] (**A**).

### 2.4. 4-Thia-15-pentadecanolide (**4**)

**Figure 4.**
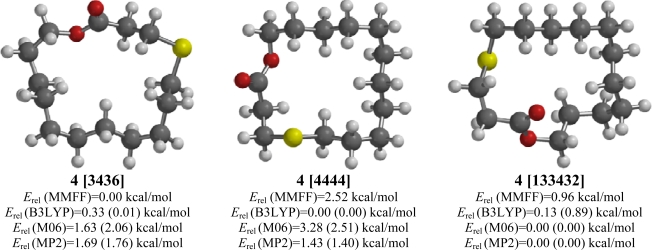
Low-energy conformations of 4-thia-15-pentadecanolide (**4**). Values in parentheses are with diffuse basis sets (6–31+G* for B3LYP and M06, 6–311+G** for MP2).

There is disagreement between the computational methods on the lowest energy conformation of 4-thia-15-pentadecanolide (**4**). Molecular mechanics (MMFF) indicate a [6343] conformation, B3LYP predict a [4444] conformation, but both M06 and MP2 show a [133432] lowest-energy conformation (Figure 4).

### 2.5. 7-Thia-15-pentadecanolide (**5**)

**Figure 5.**
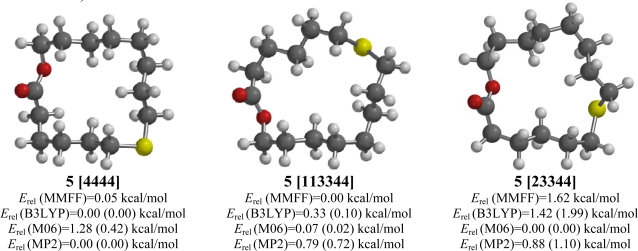
Low-energy conformations of 7-thia-15-pentadecanolide (**5**). Values in parentheses are with diffuse basis sets (6–31+G* for B3LYP and M06, 6–311+G** for MP2).

The lowest-energy conformation for cyclohexadecane has been determined to be the “square” [4444] conformation [23,24], and both B3LYP and MP2 *ab initio* calculations indicate a [4444] conformation to be the lowest energy for 7-thia-15-pentadecanolide (**5**) with a [113344] conformation only slightly higher in energy (Figure 5). Molecular mechanics (MMFF) calculations show the [4444] conformation to be 0.05 kcal/mol higher in energy than the [113344] form. The M06 method, on the other hand, calculates a [23344] conformation to be lowest energy. The placement of the sulfur atom with respect to the carbonyl group allows for a preferred *s-trans* arrangement about the ester functionality, as well as a preferred gauche arrangement for the C-S-C-C group with an exocyclic sulfur atom in the [4444] conformation.

### 2.6. 11-Thia-15-pentadecanolide (**6**)

**Figure 6.**
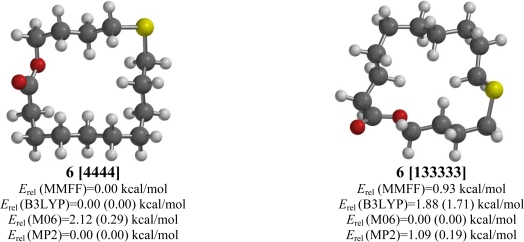
Low-energy conformations of 11-thia-15-pentadecanolide (**6**). Values in parentheses are with diffuse basis sets (6–31+G* for B3LYP and M06, 6–311+G** for MP2).

The MMFF, B3LYP, and MP2 computational methods all predict a [4444] conformation to be the lowest energy form for 11-thia-15-pentadecanolide (**6**) (Figure 6). As was the case for 7-thia-15-pentadecanolide (**5**), the location of the sulfur atom with respect to the carbonyl group allows for both the *anti* arrangement about the ester group as well as an exocyclic disposition of the sulfur. The M06 method, however, calculates the [4444] conformation to be 2.12 kcal/mol higher in energy than the lowest-energy form, a [133333] conformation.

### 2.7. 12-Thia-15-pentadecanolide (**7**)

**Figure 7.**
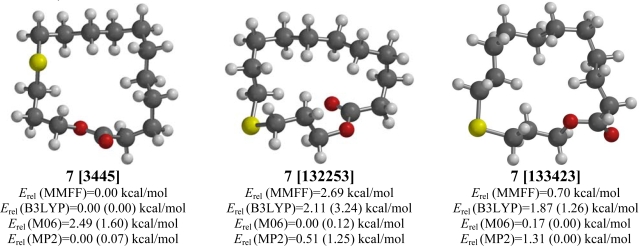
Low-energy conformations of 12-thia-15-pentadecanolide (**7**). Values in parentheses are with diffuse basis sets (6–31+G* for B3LYP and M06, 6–311+G** for MP2).

The placement of the sulfur atom at position 12 of the 16-membered ring in 12-thia-15-pentadecanolide (**7**) precludes both an *s-trans* ester group with exocyclic sulfur atom and alters the lowest-energy conformation. In this case, MMFF molecular mechanics, DFT B3LYP, and post-HF MP2 computational methods all predict a [3445] conformation (Figure 7). The DFT M06 method, however, indicates a [132253] conformation to be the lowest-energy form.

### 2.8. 6-Thia-16-hexadecanolide (**8**)

**Figure 8.**
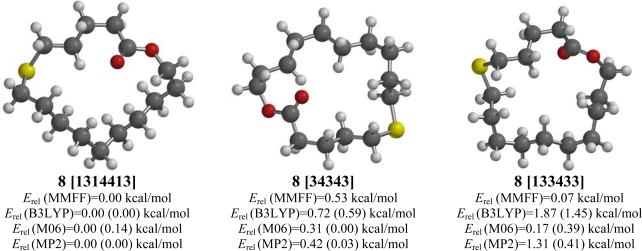
Low-energy conformations of 6-thia-16-hexadecanolide (**8**). Values in parentheses are with diffuse basis sets (6–31+G* for B3LYP and M06, 6–311+G** for MP2).

A [1314413] conformation (with pseudo two-fold symmetry) is predicted to be the lowest-energy conformation for 6-thia-16-hexadecanolide (**8**) (Figure 8). There is another low-energy conformation, a [34343] conformation (also with pseudo two-fold symmetry), however. MM2 molecular mechanics calculations have shown the [34343] conformation of cycloheptadecane to be only 0.02 kcal/mol higher in energy than the global minimum [133433] conformation [25]. The lowest-energy [133433] conformation for 6-thia-16-hexadecanolide (**8**) is also very low in energy, especially by MMFF and M06.

### 2.9. 12-Thia-16-hexadecanolide (**9**)

There is no agreement between the computational methods for the relative conformational energies of 12-thia-16-hexadecanolide (**9**). The MMFF molecular mechanics method shows a [34343] conformation to be lowest in energy, DFT B3LYP prefers a [133433] conformation, while DFT M06 predicts a [1234313] as lowest energy, and post-HF MP2 calculates a [124343] lowest in energy.

Csonka [26,27] and Truhlar [28] and co-workers have pointed out that addition of diffuse functions to a double-ζ basis set is particularly important for calculating conformational energies using density functional theory. As a check, single point calculations were carried out on the low-energy conformations of the thioether musks at the B3LYP/6–31+G*//B3LYP/6–31G*, M06/6–31+G*//M06/6–31G*, and MP2/6–311+G**//B3LYP/6–31G* levels of theory. The relative energies for 6-thia-14-tetradecanolide (**1**) showed only minor differences using the diffuse basis sets, but the trends were the same. For 10-thia-14-tetradecanolide (**2**), on the other hand, there were some changes: The [14334] conformation was the B3LYP/6–31+G* lowest-energy conformation, M06 showed a [3444] structure to be lowest in energy, while a [13353] conformation was found to be the lowest-energy MP2/6–311+G** conformation. The calculations with diffuse basis sets on 4-methyl-5-thia-14-tetra-decananolide (**3**), 4-thia-15-pentadecanolide (**4**), 7-thia-15-pentadecanolide (**5**), 11-thia-15-penta-decanolide (**6**), and 12-thia-16-hexadecanolide (**9**) showed only minor differences. With the diffuse basis sets, the lowest-energy conformation for 12-thia-15-pentadecanolide (**7**) for both M06 and MP2 is the [133423], but MP2 still shows the [3445] conformation to be very low energy (*E*_rel_=0.07 kcal/mol), while the M06 method shows the [132253] form to be low in energy (*E*_rel_=0.12 kcal/mol). In 6-thia-16-hexadecanolide (**8**) only the M06 method showed a difference with the diffuse basis set, calculating the [34343] conformation to be 0.14 kcal/mol lower in energy than the [1314413].

Unfortunately, there are no experimental data (*i.e.*, X-ray crystal structures) of thioether musks available in order to compare structural parameters of the DFT methods. However, the crystal structure of 3-methyl-1,5,9-trithiacyclododecane-3-carboxylic acid (**10**) [29], is available. A comparison of average structural parameters for this compound as well as 6-thia-14-tetradecanolide (**1**), [13434] conformation, and 7-thia-15-pentadecanolide (**5**), [4444] conformation, is presented in Table 1. Comparison of the structural parameters for macrocycle **10** indicates that M06 generally gives more accurate bond lengths than B3LYP. Bond angles around carbon are also more accurate for M06 compared to B3LYP, but C-S-C bond angles are better modeled by B3LYP. The M06 method generally gives shorter bonds and more acute bond angles than B3LYP. Comparing ring torsion angles in macrocycle **10**, B3LYP had an average deviation of 2.41° while M06 torsion angles deviated an average of 2.15°. Thus, for macrocyclic thioethers, M06 generally gives more accurate structural parameters than B3LYP (at least at the 6–31G* level).

Although the thioether musks are relatively non-polar molecules, it might be expected that solvation could drastically change the relative conformational energies for these macrocyclic systems. The energies of the low-energy conformations were re-evaluated using the empirical SM5.4 aqueous solvation model (Table 2). In most cases there was little change, but in some cases, aqueous solvation resulted in a change in relative conformational energies.

## 3. Computational Methods

All calculations were carried out using SPARTAN’08 for Windows [30]. Initial conformational analyses were carried out on each macrocycle using a Monte-Carlo molecular mechanics conformational search using the MMFF force field [10]. For each macrocycle, all conformations with *E*_rel_ less than 3 kcal/mol from the MMFF conformational analysis were then modeled using both density functional theory and Hartree-Fock and post-HF methods. Both the popular B3LYP [11,12] and the recently developed M06 [13] functionals and the 6-31G* basis set [31] were used for the optimization of all stationary points in the gas phase. Single-point Hartree-Fock *ab initio* energies were calculated using the DFT geometries (above) at the 6–31G** [31] level, followed by a correlation energy calculation using the second-order Møller-Plesset model (MP2) [31]. In addition, single point calculations were carried out on the low-energy conformations of the thioether musks at the B3LYP/6–31+G*//B3LYP/6–31G*, M06/6–31+G*//M06/6–31G*, and MP2/6–311+G**//B3LYP/6–31G* levels of theory. All enthalpies are zero-point (ZPE) corrected with unscaled frequencies, but with no thermal corrections; they are, therefore, *H*_(0K)_. Relative energies (*E*_rel_) were calculated from the *H*_(0K)_ values. Aqueous solvation energies were determined using the empirical SM5.4 model [32].

## 4. Conclusions

The results from this study indicate that conformationally mobile macrocyclic ring systems remain difficult to computationally model, even with relatively large basis sets and diffuse functions. At the levels of theory used in these current calculations, there seems to be little agreement between the two DFT methods (B3LYP and M06) as well as with the MP2 *ab initio* method (Table 2). With new and improving functionals, larger basis sets, and increased computational power, this situation will hopefully improve.

## Figures and Tables

**Figure 9.**
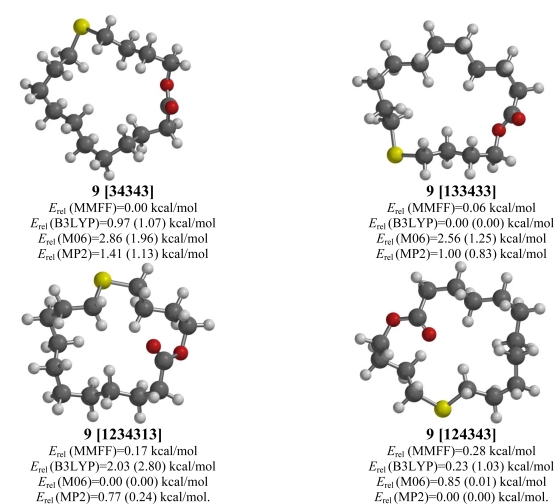
Low-energy conformations of 12-thia-16-hexadecanolide (**9**). Values in parentheses are with diffuse basis sets (6–31+G* for B3LYP and M06, 6–311+G** for MP2).

**Scheme 1.**
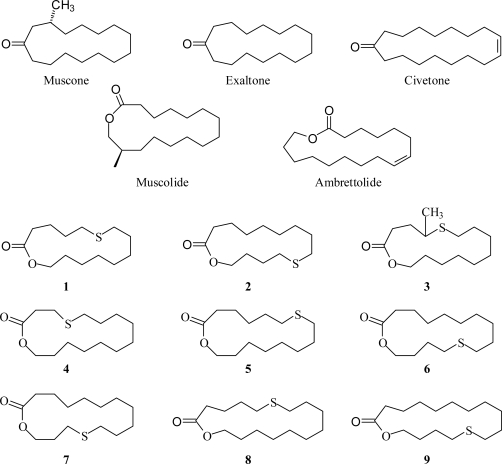
Macrocyclic musks discussed in this work.

**Table 1.** Comparison of structural parameters for B3LYP and M06 for **10**, **1** [13434], and **5** [4444]. 
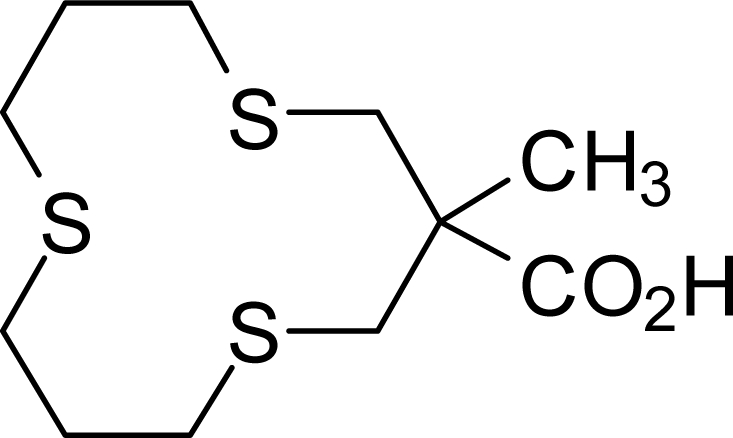

**Bond Lengths (Å)**	**X-ray**	**10**	**M06**	**1 [13434]**		**5 [4444]**	
		**B3LYP**		**B3LYP**	**M06**	**B3LYP**	**M06**
CH_2_–CH_2_	1.522	1.539	1.525	1.536	1.522	1.536	1.522
CH_2_–S (endodentate)	1.816	1.845	1.833	1.843	1.829	---	---
CH_2_–S (exodentate)	1.798	1.839	1.827	---	---	1.841	1.828
C=O	1.206	1.211	1.206	1.214	1.209	1.213	1.209
C–O	1.316	1.356	1.346	1.353	1.344	1.354	1.345
Bond Angles (deg)							
CH_2_–CH_2_–CH_2_	112.5	112.9	112.4	114.12	113.48	113.95	113.32
CH_2_–CH_2_–S	113.9	114.4	113.8	116.0	115.8	115.8	115.4
CH_2_–S–CH_2_ (endodentate)	100.1	100.1	98.4	103.1	101.6	---	---
CH_2_–S–CH_2_ (exodentate)	101.1	101.4	100.5	---	---	102.9	101.3
O–C=O	122.7	122.2	122.4	124.0	124.3	123.9	123.9

**Table 2.** Summary of low-energy conformations (including aqueous solvation) for thioether musks.

**Compound** ** (Conformation)**	**Dipole** **(*D*)**	***E*_rel_ (kcal/mol)^a^**			
		**MMFF**	B3LYP/6–31+G*	M06/6–31+G*	MP2/6–311+G**
6-Thia-14-tetradecanolide (**1**)					
[13434]	0.88	0.00	0.00 (0.00)	0.49 (0.00)	0.64 (0.58)
[13353]	1.90	0.49	1.33 (1.40)	0.48 (0.04)	0.00 (0.00)
[23343]	1.15	0.10	0.27 (0.74)	0.00 (0.00)	0.78 (1.19)

10-Thia-14-tetradecanolide (**2**)					
[13434]	0.76	0.00	0.60 (0.73)	0.42 (0.70)	1.22 (1.38)
[13353]	1.94	0.26	1.17 (1.14)	0.36 (0.51)	0.00 (0.00)
[23343]A	3.11	1.24	0.16 (0.54)	1.01 (1.68)	1.33 (1.74)
[23343]B	0.77	0.20	0.38 (0.95)	0.23 (1.01)	1.06 (1.65)
[14334]	1.73	0.54	0.00 (0.00)	0.37 (0.56)	0.38 (0.41)
[3444]	1.35	1.22	0.43 (0.20)	0.00 (0.00)	1.41 (1.21)

4-Methyl-5-thia-14-tetradecananolide (**3**)					
[3444]A	0.91	0.00	0.75 (0.89)	0.00 (0.00)	0.00 (0.00)
[3444]B	0.97	0.54	0.70 (0.65)	0.67 (0.41)	0.43 (0.24)
[14334]	1.60	0.28	0.00 (0.00)	0.81 (0.83)	0.81 (0.67)

4-Thia-15-pentadecanolide (**4**)					
[4444]	3.19	2.52	0.00 (0.00)	2.51 (2.22)	1.40 (0.94)
[3436]	3.51	0.00	0.01 (0.01)	2.06 (1.90)	1.76 (1.30)
[133432]	0.65	0.96	0.89 (1.34)	0.00 (0.00)	0.00 (0.00)

7-Thia-15-pentadecanolide (**5**)					
[4444]	2.43	0.05	0.00 (0.00)	0.42 (0.00)	0.00 (0.00)
[23344]	1.55	1.62	1.99 (3.01)	0.00 (0.77)	1.10 (2.12)
[113344]	1.54	0.00	0.10 (0.75)	0.02 (0.21)	0.72 (1.36)

11-Thia-15-pentadecanolide (**6**)					
[4444]	1.81	0.00	0.00 (0.00)	0.29 (0.00)	0.00 (0.00)
[133333]	2.25	0.93	1.71 (2.82)	0.00 (0.81)	0.19 (1.31)

12-Thia-15-pentadecanolide (**7**)					
[3445]	3.27	0.00	0.00 (0.00)	1.60 (2.22)	0.07 (0.57)
[132253]	2.49	2.69	3.24 (3.16)	0.12 (0.50)	1.25 (1.67)
[133423]	2.44	0.70	1.26 (0.76)	0.00 (0.00)	0.00 (0.00)

6-Thia-16-hexadecanolide (**8**)					
[34343]	2.87	0.53	0.59 (0.46)	0.00 (0.00)	0.03 (0.00)
[133433]	3.20	0.07	1.45 (1.21)	0.39 (0.49)	0.41 (0.26)
[1314413]	2.24	0.00	0.00 (0.00)	0.14 (0.38)	0.00 (0.11)

12-Thia-16-hexadecanolide (**9**)					
[34343]	1.77	0.00	1.07 (0.93)	1.96 (1.42)	1.13 (0.61)
[133433]	1.51	0.06	0.00 (0.00)	1.25 (0.56)	0.83 (0.16)
[124343]	2.76	0.28	1.03 (1.71)	0.01 (0.00)	0.00 (0.00)
[1234313]	2.78	0.17	2.80 (3.72)	0.00 (0.22)	0.24 (0.48)

^a^ Relative energies including aqueous solvation are in parentheses.
